# Area-dependent time courses of brain activation during video-induced symptom provocation in social anxiety disorder

**DOI:** 10.1186/2045-5380-4-6

**Published:** 2014-04-28

**Authors:** Stephanie Boehme, Alexander Mohr, Michael PI Becker, Wolfgang HR Miltner, Thomas Straube

**Affiliations:** 1 Department of Biological and Clinical Psychology, Friedrich Schiller University Jena, Am Steiger 3 // 1, Jena D-07743, Germany; 2 Institute of Medical Psychology and Systems Neuroscience, University of Muenster, Von-Esmarch-Str. 52, Muenster D-48149, Germany

**Keywords:** Social anxiety disorder, Symptom provocation, Functional magnetic resonance imaging (fMRI), Amygdala, Insula, Medial prefrontal cortex

## Abstract

**Background:**

Previous functional imaging studies using symptom provocation in patients with social anxiety disorder (SAD) reported inconsistent findings, which might be at least partially related to different time-dependent activation profiles in different brain areas. In the present functional magnetic resonance imaging study, we used a novel video-based symptom provocation design in order to investigate the magnitude and time course of activation in different brain areas in 20 SAD patients and 20 healthy controls.

**Results:**

The disorder-related videos induced increased anxiety in patients with SAD as compared to healthy controls. Analyses of brain activation to disorder-related *versus* neutral video clips revealed amygdala activation during the first but not during the second half of the clips in patients as compared to controls. In contrast, the activation in the insula showed a reversed pattern with increased activation during the second but not during the first half of the video clips. Furthermore, a cluster in the anterior dorsal anterior cingulate cortex showed a sustained response for the entire duration of the videos.

**Conclusions:**

The present findings suggest that different regions of the fear network show differential temporal response patterns during video-induced symptom provocation in SAD. While the amygdala is involved during initial threat processing, the insula seems to be more involved during subsequent anxiety responses. In accordance with cognitive models of SAD, a medial prefrontal region engaged in emotional-cognitive interactions is generally hyperactivated.

## Background

Individuals suffering from social anxiety disorder (SAD), classified as ‘social phobia’ in DSM-IV-TR [1], show exaggerated fear responses in social or performance situations. In particular, patients are excessively concerned about being evaluated negatively by others. In search of the neural basis of SAD, different brain areas have been identified that seem to be involved in SAD. By means of functional brain imaging, heightened activation of the amygdala has been found during the processing of disorder-related stimuli (for example, [2-9]) as well as during symptom provocation in SAD patients (for example, [10-14]), supporting the assumed role of the amygdala in threat processing [15,16]. Furthermore, several other regions have been associated with increased activation in SAD, including medial prefrontal areas, for example, dorsal anterior cingulate cortex (ACC) and dorsomedial prefrontal cortex (dmPFC), and the insular cortex (for example, [3,5,8,10,17-20]). Medial prefrontal cortex areas have been proposed to be linked to explicit emotional evaluation, emotional-cognitive interactions, self-referential processing, and emotion-regulation [21-26]. The insula seems to be involved in interoception and representation of bodily states [27-29] and might support aversive feelings by evaluating arousal responses [28,30,31].

However, although these areas have been repeatedly shown to be associated with the processing of disorder-relevant stimuli in SAD and other anxiety disorders [32], reported brain activation patterns are rather inconsistent across studies with most studies describing different areas to be involved. Furthermore, there are only few symptom provocation studies as compared to the large number of studies that investigated the neural correlates during the processing of social stimuli such as facial expressions in SAD patients. Remarkably, even though disorder-related stimuli such as emotional faces do not induce reliable anxiety symptoms in patients, they seem to activate parts of an emotional network. However, findings are variable and strongly depend on task conditions [8,9] and time course parameters [18,33].

Reliable anxiety responses are induced by symptom provocation designs such as actual or anticipated public performance. Furthermore, findings from anxiety symptom provocation studies should provide stronger evidence which regions are involved in anxiety symptoms in SAD. While some symptom provocation studies reported increased amygdala activation during public speaking in patients with SAD [11-14,34], studies using other symptom provocation tasks did not [35-37]. Similarly, there are also inconsistencies regarding the involvement of the insula (see [10,12,13,35-38]) and prefrontal regions in SAD [12-14,34,36,37].

Obviously, threat-related brain activation in SAD depends on various factors, which are not well understood yet. For example, some symptom provocation tasks such as overt speaking tasks are associated with active performance but are also inherently susceptible to brain imaging-relevant artefacts such as head movements and performance differences between patients and controls. Moreover, in different tasks, different functions of the threat-processing network might be involved. Furthermore and most importantly, brain activation was shown to vary over time in response to anticipatory anxiety in social anxiety (see [10]) and some variability in previous findings may be due to different time courses of brain activation. Accordingly, there is general evidence that indicates different time courses of several brain areas within the defense cascade (for example, [39,40]). Thus, while the amygdala has been suggested to be primarily relevant during the initial period of threat processing in healthy participants and patients with phobias (for example, [39-42]), the insula and prefrontal areas were shown to be associated with explicit and more sustained fear responses [39,40,42-44]. In SAD, the time course of activation in different brain areas during symptom provocation is largely unknown. A recent study found increased amygdala activation only during the first half of an anticipatory threat interval in SAD [10].

In the present study, we used a novel symptom provocation design in SAD by presenting disorder-related and neutral video clips. We developed a new set of video stimuli for symptom provocation in SAD, based on evidence that the use of short film clips represents one of the most effective and reliable methods to induce emotions in laboratory settings [45-47]. The study aimed to investigate increased brain activation in several areas that have been identified to be important in SAD during symptom provocation (amygdala, insula, ACC, and dmPFC). Activation was modelled to account (a) for the full time course of the video clips, and (b) specifically, for the first and (c) second half of the clips. If the amygdala bears specific relevance for initial threat processing, effects should be most pronounced during the first half of the video clips. In contrast, responses in other areas should also be manifest during the second half of the video clips or may occur specifically during the second half of the clips.

## Methods

### Participants

Twenty-one patients with a primary diagnosis of SAD of the generalized subtype and 20 healthy control participants (HC) took part in the study. Due to strong head movement (>3 mm) one patient had to be excluded from analyses. Therefore, the final sample comprised 20 SAD and 20 HC participants. All were right-handed with normal or corrected-to-normal vision. They were recruited via public announcement and provided written informed consent for participation. The study was approved by the ethics committee of the University of Jena. Diagnoses were confirmed by clinical psychologists administering the Structured Clinical Interview for DSM-IV Axis I and II disorders (SCID I and II [48,49]). Exclusion criteria were any of the following: (1) A diagnosis of panic disorder and/or agoraphobia, current alcohol/substance abuse, psychotic disorder, dementia, primary or secondary major depression; (2) a history of seizures or head injury with loss of consciousness; (3) a severe uncontrollable medical condition; and (4) the use of any psychotropic medication within the preceding 6 months. HC were free of any psychopathology and medication. In the SAD sample, co-morbidities were specific phobia (n = 3), obsessive-compulsive disorder (n = 1), bulimia nervosa (recurrent in full remission; n = 1), and depressive episodes in the past (n = 5). Six patients also met the criteria of an Axis II personality disorder (anxious (avoidant) personality disorder, dependent personality disorder). Patients with SAD and HC participants were matched for age (SAD: 23.85 years, HC: 24.20 years, *t*[38] = 0.45, *P* >0.05), gender (SAD: 10 women, HC: 10 women, *χ*^*2*^[1] = 0.00, *P* >0.05) and education (all participants had high school graduation with a minimum school period of 12 years). Before scanning, all participants completed the LSAS (Liebowitz Social Anxiety Scale, German version, [50]) and the BDI (Beck Depression Inventory, German version, [51]) questionnaire. SAD patients scored significantly higher on both LSAS (SAD: LSAS = 71.95, HC: LSAS = 10.65, *t*[38] = 18.23, *P* <0.05) and BDI (SAD: BDI = 11.90, HC: BDI = 3.05, *t*[38] = 8.33, *P* <0.05) questionnaires than HC participants.

### Paradigm

Stimuli consisted of disorder-related (social) and disorder-unrelated (neutral) video clips that lasted 24 s each. The clips were developed by our group and filmed with the help of experienced actors who belonged to student or layman theater groups. The clips showed a man or woman (counter-balanced) acting either in a social (social activity) or in a corresponding neutral situation (same environment but actor is alone and engaged in a non-social activity). Prototypically feared situations for the generalized subtype of SAD were subsumed in four broad categories: (1) formal interaction situations (for example, oral examinations); (2) informal interaction situations (for example, asking someone for directions); (3) situations that require self-assurance (for example, complaints about goods); and (4) situations where the actor’s behavior is observed by others (for example, during social eating; see Additional file 1: Table S1: Description of the used video clips). Eighteen disorder-related and 18 neutral video clips were chosen from an initial pool of 36 social and 36 neutral clips by five leading German experts on SAD with extensive experience in diagnosis and therapy of SAD (see Acknowledgments) who judged the anxiety-inducing potential and social phobia-relevance of the clips on nine-point Likert scales. Based on these ratings, a final set of maximally anxiety-inducing and SAD-related videos was chosen which comprised five videos for the categories (1) and (4) and four videos for the categories (2) and (3), respectively. All disorder-related videos had to exceed a rating cutoff score of κ_s_ = 5 and neutral videos had to fall below κ_s_. On average, phobia-relevance of disorder-related videos used in the present study was rated M = 7.10 (SD = ±.52), and anxiety-inducing potential was rated M = 7.03 (SD = ±.81), while neutral videos were rated only minimally anxiety-inducing (M = 2.10 (SD = ±0.54)) and phobia-relevant (M = 2.04 (SD = ±0.52)). The order of clips was pseudo-randomized with no more than two clips of the same category (social or neutral) succeeding each other. Inter-stimulus interval (white fixation cross in front of a black screen) was set to 16 s. Participants were asked to focus on the main actor of the scene, to take his/her perspective and to empathize as much as possible with her/his behavior.

After magnetic resonance imaging (MRI), participants were re-exposed to the clips and were asked to rate valence, arousal, and anxiety which were induced by each clip on a nine-point Likert scale (valence: 1 = very pleasant to 9 = very unpleasant, whereas 5 = neutral; arousal: 1 = not arousing to 9 = very arousing; anxiety: 1 = not anxious to 9 = very anxious). Behavioral data were analyzed by repeated measures analyses of variance (ANOVA) and subsequent t-tests using SPSS software (Version 19.0.0.1, SPSS, Inc.). For ANOVAs and t-tests a probability level of *P <*0.05 was considered statistically significant.

### Functional magnetic resonance imaging

Scanning was performed in a 1.5 Tesla magnetic resonance scanner (‘Magnetom VISION Plus’, Siemens, Medical Solutions, Erlangen, Germany). After a T1-weighted anatomical scan, two runs with 184 volumes (each video clip appeared once in a run) were conducted using a T2*-weighted echo-planar sequence (TE, 50 ms; flip angle, 90°; matrix, 64 × 64; field of view, 192 mm; TR, 3.9 s). Each volume consisted of 40 axial slices (thickness, 3 mm; gap, 0 mm; in plane resolution, 3 × 3 mm). The first four volumes were discarded from analysis to ensure steady-state tissue magnetization.

Functional magnetic resonance imaging (fMRI) data preprocessing and analyses were realized by BrainVoyager QX software (Version 1.10.4; Brain Innovation BV). As a first step of preprocessing, all volumes were realigned to the first volume in order to minimize artifacts due to head movements. Afterwards, spatial (8 mm full-width half-maximum isotropic Gaussian kernel) and temporal filter were applied (high pass filter: 3 cycles per run; low pass filter: 2.8 s; linear trend removal). Then, the anatomical and functional images were co-registered and normalized to the Talairach space [52].

Statistical analyses of blood oxygen-level-dependent (BOLD) data were performed by multiple linear regression of its signal time course at each voxel. The expected signal change of BOLD response for each event type (predictor) was modeled by a canonical hemodynamic response function. First, the whole duration intervals of the video clips were defined as predictors. Second, for investigating the time course of activation, the period of brain activation to social and neutral video clips was divided into two succeeding parts of 12 s each and a new general linear model (GLM) was computed. Both GLMs comprised motion correction parameters as events of no interest. Statistical comparisons were realized using a mixed effect analysis, which considers inter-subject variance and permits population-level inferences. Then, voxel-wise statistical maps were generated and the relevant, planned contrasts of predictor estimates (beta-weights) were computed for each individual. After that, a random effects group analysis of these individual contrasts was performed.

First, analyses were conducted for specific regions of interest (ROIs). Following the approach recommended by Eickhoff et al. [53], we extracted the amygdala ROI consisting of three bilateral amygdala maximum probability maps (laterobasal, centromedial, and superficial; 9,077 mm^3^ in total) of the anatomy toolbox [54]. ROIs for the bilateral insula (32,822 mm^3^), ACC (23,963 mm^3^), and dmPFC (medial division of the superior frontal cortex; 44,945 mm^3^) were extracted from the AAL atlas included in WFU PickAtlas software [55-57]. Using MATLAB (Version 7.8; The MathWorks, Inc) all maps were transformed into BrainVoyager-compatible Talairach coordinates via ICBM2tal [58]. Second, whole brain analyses were conducted.

Statistical parametric maps resulting from voxel-wise analyses were considered statistically significant for clusters that survived a correction for multiple comparisons. For this purpose, we used the approach as implemented in BrainVoyager (based on a 3D extension of the randomization procedure described by Forman et al. [59]). First, voxel-level threshold was set to *P* <0.005 (uncorrected) for the ROI-based and to *P* <0.001 (uncorrected) for the whole brain analyses. Then, threshold maps were submitted to a correction for multiple comparisons that was firstly calculated for each ROI and secondly for the whole brain. The correction is based on the estimation of the cluster threshold that is the minimal number of voxels necessary to control for multiple comparisons. The cluster threshold criterion was based on an estimate of each map’s spatial smoothness [59] and on an iterative procedure (Monte Carlo simulation). The Monte-Carlo simulation used 1,000 iterations in order to estimate the minimum cluster size threshold that yielded a cluster-level false-positive rate of 5%. The cluster size thresholds (full length: amygdala, 88 mm^3^; insula, 180 mm^3^; ACC, 142 mm^3^; dmPFC, 167; first and second half: amygdala, 79 mm^3^; insula, 162 mm^3^; ACC, 108 mm^3^; dmPFC, 156 mm^3^) were applied to the statistical maps. Finally, activation of peak voxels in the ROIs was correlated with symptom severity as measured by LSAS. For this purpose SPSS was used.

## Results

### Rating data

**Figure 1:**
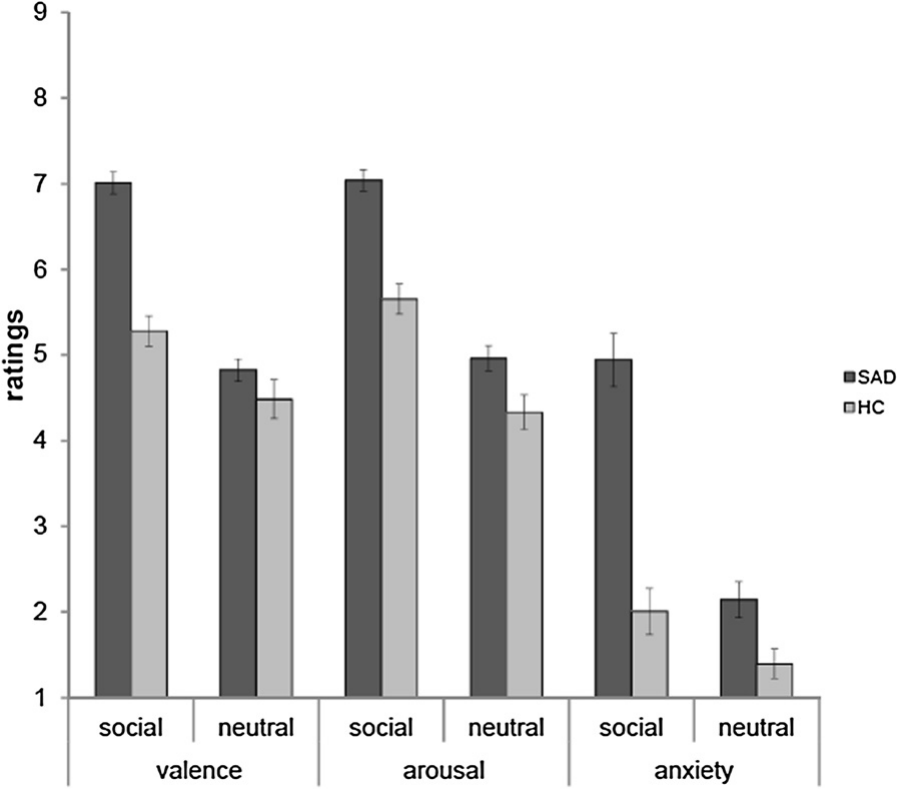
Valence, arousal, and anxiety ratings (mean ± standard error) for social and neutral video clips in patients with social anxiety disorder (SAD) and healthy control participants (HC).

Analyses of post scanning stimulus ratings showed that both SAD patients and HC participants rated social video clips as more negative (*F*[1,38] = 170.61, *P* <0.05), more arousing (*F*[1,38] = 222.71, *P* <0.05), and more anxiety-inducing (*F*[1,38] = 185.69, *P* <0.05) than neutral video clips. Additionally, SAD patients as compared to controls rated all video clips as more unpleasant (F[1,38] = 24.23, *P* <0.05), more arousing (F[1,38] = 24.68, *P* <0.05), and anxiety inducing (F[1,38] = 32.97, *P* <0.05). Furthermore, there was a significant group by condition interaction (valence: *F*[1,38] = 37.65, *P* <0.05; arousal: *F*[1,38] = 11.16, *P* <0.05; anxiety: *F*[1,38] = 76.46, *P* <0.05) with increased ratings for social *versus* neutral video clips in SAD patients as compared to HC participants. Figure 1 shows rating data for SAD and HC participants.

### fMRI data

#### Interaction group by video valence

**Figure 2:**
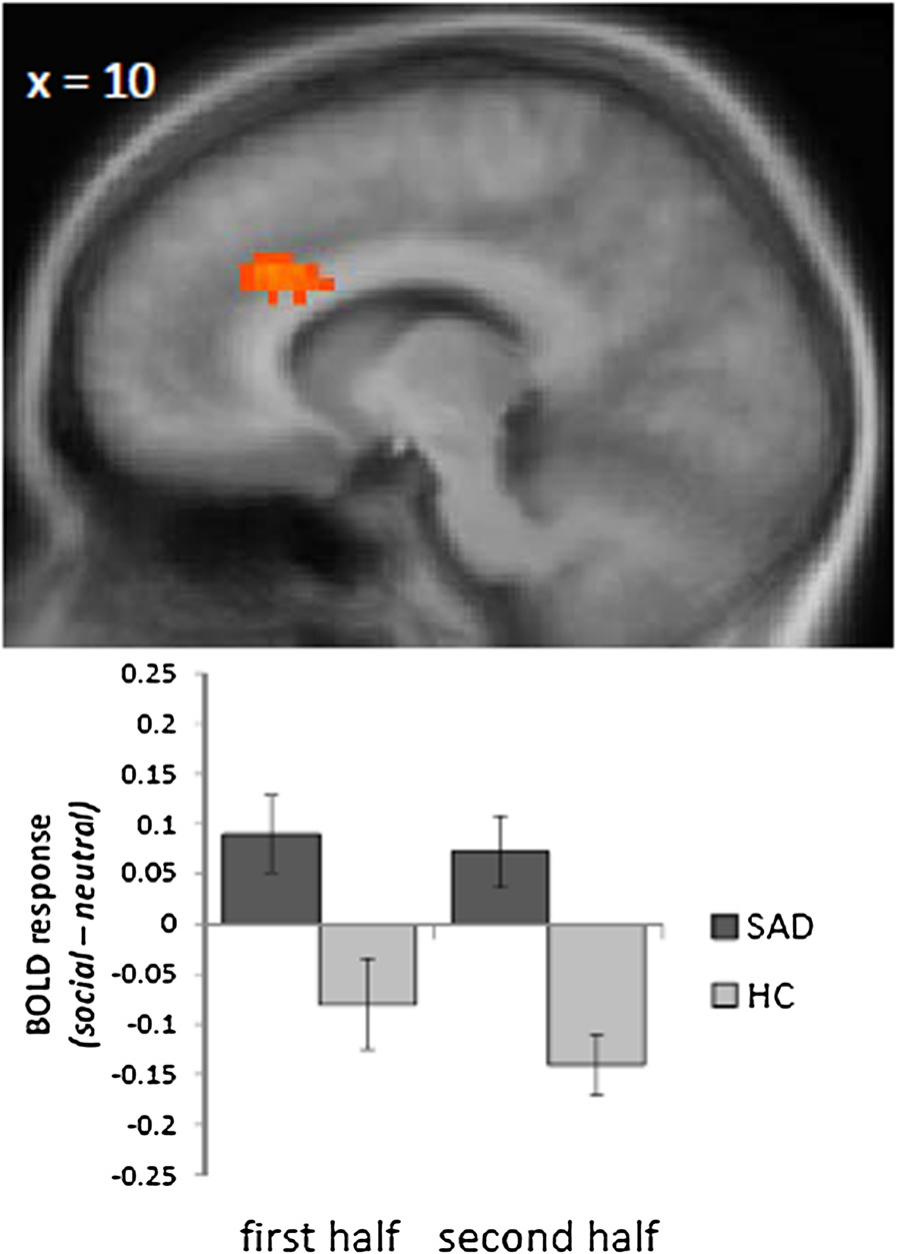
**Differential brain activation in the anterior dorsal ACC during the social *vs. *neutral video clip presentation.** Patients with social anxiety disorder (SAD) displayed an enhanced activation as compared to healthy control participants (HC) during the first as well as during the second part of the video clips (social > neutral). Statistical parametric maps are overlaid on a T1 scan (radiological convention: left = right). The plot at the bottom displays contrasts of parameter estimates (social *vs.* neutral video clips for first and second half separately; mean ± standard error for maximally activated voxel).

We investigated BOLD activation during the full length of video clips and during the first and second period of clip presentation. When analyzing the full length of the social *versus* neutral video clips in SAD as compared to HC participants, we only detected significant activation differences in the prefrontal cortex. There was a cluster of activated voxels in the right anterior dorsal ACC (peak voxel Talairach coordinates: x = 14; y = 20; z = 28; size = 1,026 mm^3^; *t*[38] = 4.45; see Figure 2).

**Figure 3:**
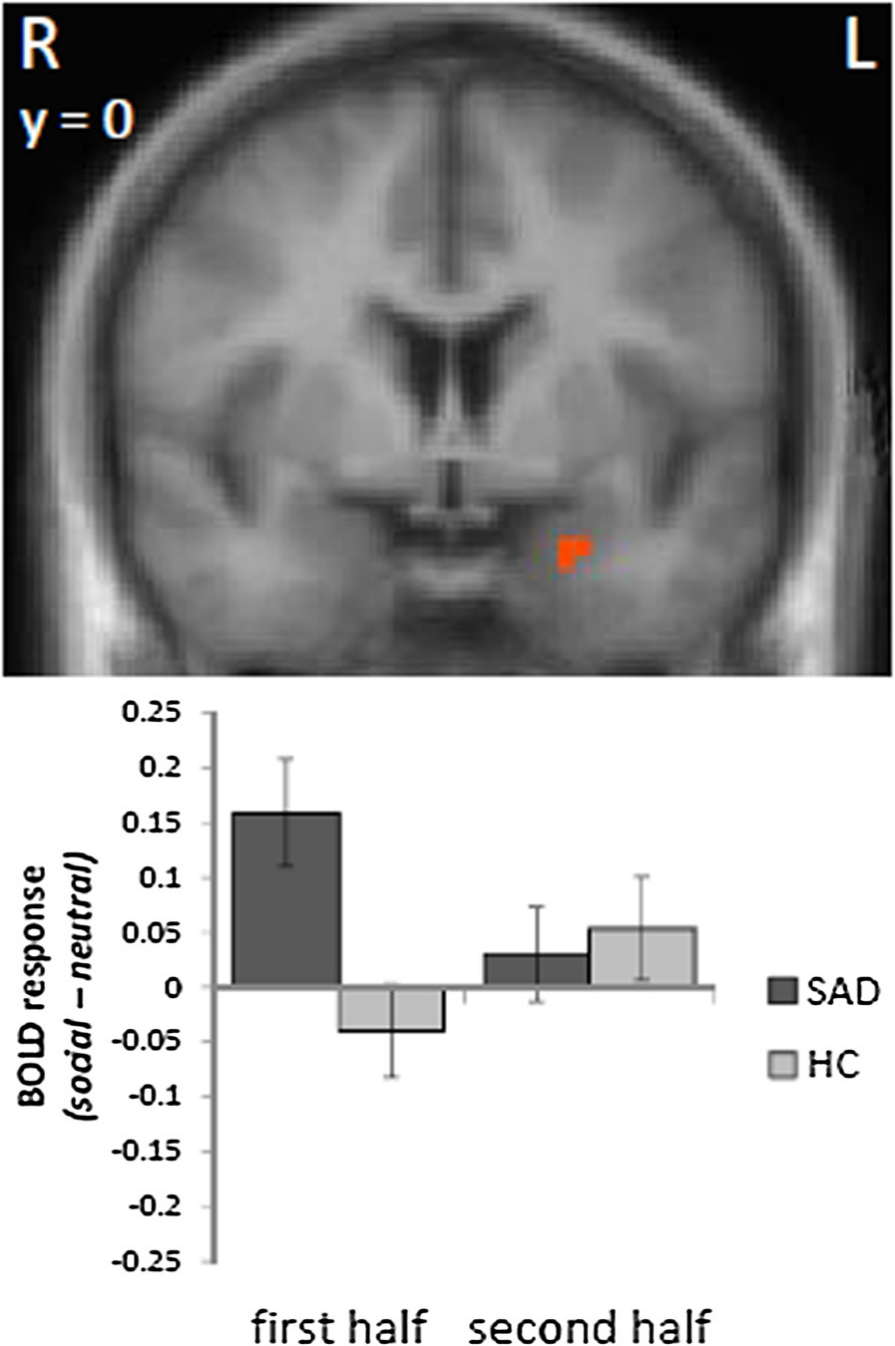
**Differential brain activation during the first half of the social *vs. *neutral video clips.** Patients with social anxiety disorder (SAD) displayed an enhanced activation in the left amygdala as compared to healthy control participants (HC; social > neutral video clips). Statistical parametric maps are overlaid on a T1 scan (radiological convention: left = right). The plot shows contrasts of parameter estimates (social *vs.* neutral video clips for first and second half separately; mean ± standard error for maximally activated voxel).

**Figure 4:**
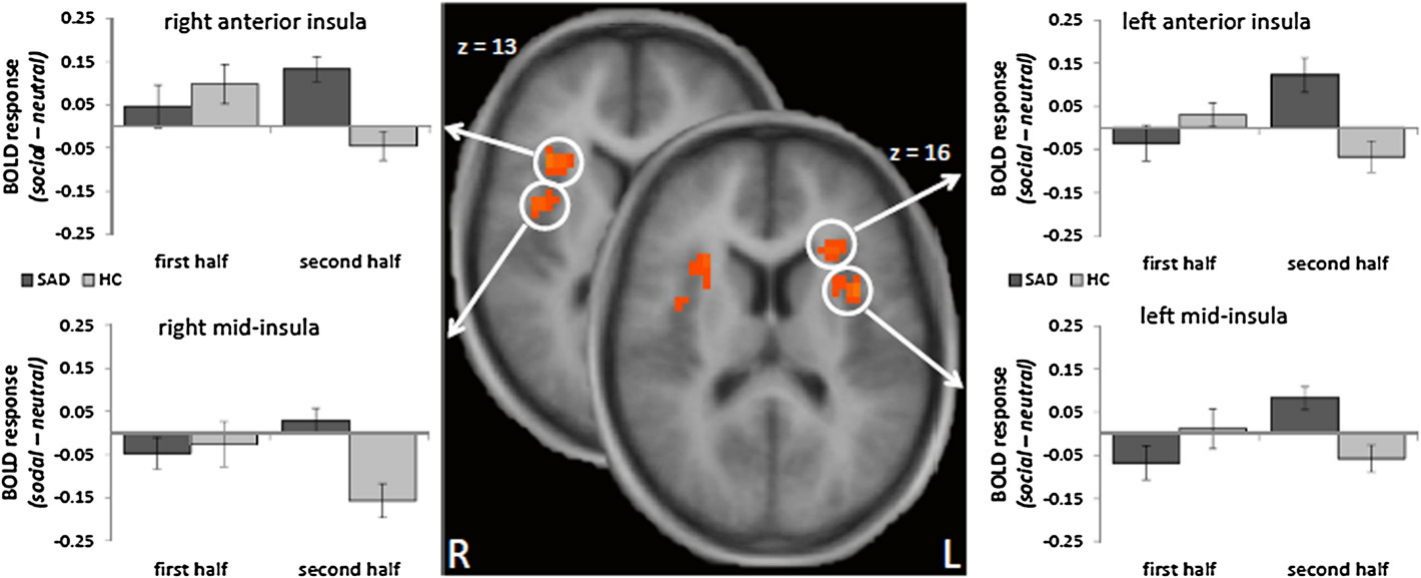
**Differential brain activation during the second half of the social *vs. *neutral video clips.** Patients with social anxiety disorder (SAD) displayed enhanced activation within the left and right anterior and mid-insula as compared to healthy control participants (HC; social > neutral video clips). Statistical parametric maps are overlaid on a T1 scan (radiological convention: left = right). The bar graphs show contrasts of parameter estimates (social *vs.* neutral video clips for first and second half separately; mean ± standard error for maximally activated voxel).

However, when analyzing BOLD activation during the first and second half of the video clips separately, we observed a hyperactivation of the left amygdala in response to social *versus* neutral video clips during the first half of the video clips in SAD patients as compared to HC participants (peak voxel Talairach coordinates: x = -23; y = 0; z = -19; size = 81 mm^3^; *t*[38] = 2.93; probability = 50%; see Figure 3). In contrast, activation in the left insula differed significantly during the second half of the social *versus* neutral video clips in SAD as compared to HC participants. There were two clusters of hyperactivated voxels in the left (anterior cluster: peak voxel Talairach coordinates: x = -24; y = 23; z = 13; size = 756 mm^3^; *t*[38] = 3.61; mid-insula cluster: peak voxel Talairach coordinates: x = -36; y = 5; z = 16; size = 648 mm^3^; *t*[38] = 4.31; see Figure 4) and in the right insula (anterior cluster: peak voxel Talairach coordinates: x = 36; y = 20; z = 13; size = 999 mm^3^; *t*[38] = 4.11; mid-insula cluster: peak voxel Talairach coordinates: x = 42; y = -1; z = 13; size = 324 mm^3^; *t*[38] = 3.83; see Figure 4) for social *versus* neutral video clips during the second half in SAD *versus* HC subjects.

Furthermore, a cluster in the anterior dorsal ACC was found to be stronger activated in SAD *versus* HC participants during both halves of the social *versus* neutral video clips. The clusters were almost at the same location with similar peak voxels (first half: peak voxel Talairach coordinates: x = 14; y = 21; z = 29; size = 108 mm^3^; *t*[38] = 3.22; second half: peak voxel Talairach coordinates: x = 9; y = 27; z = 29; size = 1,431 mm^3^; *t*[38] = 4.44). Within the ROIs, there were no clusters of greater activation during neutral > social video clips in SAD *versus* HC subjects. For the sake of completeness, results of the whole brain analysis are shown in Table 1, indicating primarily additional increased activations in SAD patients in (pre)frontal cortex during both halves of the videos.

**Table 1:** Whole brain analysis of group differences in activation between social and neutral videos (SAD > HC)

		**Social > neutral**					**Neutral > social**				
	**Hemisphere**	**Talairach**			**t-value**	**Size (mm**^**3**^**)**	**Talairach**			**t-value**	**Size (mm**^**3**^**)**
		**x**	**y**	**z**			**x**	**y**	**z**		
*Whole video*											
Superior frontal gyrus (BA 10)	R	23	62	27	4.54	621					
*First half*											
Globus pallidus	R						15	-1	6	4.49	216
*Second half*											
Middle frontal gyrus (BA 46)	L	-50	24	19	3.66	297					
Inferior frontal gyrus (BA 44)	R	57	11	10	4.52	1350					
Superior frontal gyrus (BA 8)	L	-15	50	39	4.18	513					
Superior frontal gyrus (BA 9)	R	19	60	24	4.54	1215					
Inferior parietal gyrus (BA 40)	R						55	-48	42	3.64	162
Inferior temporal gyrus (BA 20)	R						46	-7	-34	3.77	567

Peak coordinates obtained at a threshold of *P* <0.001 and cluster size ≥143 mm^3^ voxels.

BA: Brodmann Area.

#### Correlational analysis

Finally, correlations between activation of significant peak voxels within the ROIs and symptom severity in SAD as measured by LSAS was investigated. This revealed no significant correlation in SAD patients (for all analyses *P* >0.05).

## Discussion

The present study investigated brain activation in response to disorder-related and anxiety-provoking video clips *versus* neutral video clips in patients with SAD and healthy controls. Results showed that brain activation varies over time during symptom provocation in SAD as compared to HC subjects. The left amygdala was hyperactivated in SAD patients compared to controls specifically during the first part of the disorder-related video clips. Specifically during the second part of the video clips, SAD patients showed stronger insula activation than controls in response to social *versus* neutral video clips. Finally, increased activation of the anterior dorsal ACC to social *versus* neutral video clips was found during the whole time course of video presentation in patients with SAD compared to HC participants.

The hyperactivation of the amygdala during disorder-related video clips in SAD is in accordance with previous studies that reported increased amygdala responses during threat processing in SAD patients (for example, [3-8,60-65]; but see [20,35-37,66,67]). The amygdala, due to its interconnections to various cortical regions and to the brain stem and the hypothalamus additionally, is suggested to be of essential relevance for mediation of automatic, bottom-up processing of emotional, and particularly threatening stimuli [15,68-70]. Furthermore, the present amygdala hyperactivation in SAD patients was found during the first half of the video presentation only. This implies a temporally restricted role of the amygdala at least during some forms of symptom provocation in SAD. The current finding is in accordance with a recent study on anticipatory anxiety in social anxiety [10] and allocates the amygdala a central role within a transient threat detection system [71,72], which affects both regulation of the autonomic nervous system as well as modulation of perceptual and emotional processing of relevant stimuli [9,68-70,73].

Repeatedly, the insula was shown to be involved in the processing of aversive emotional cues in SAD and other anxiety disorders [32]. Especially the anterior insula has been shown to play an important role in the processing of visceral and autonomic responses to emotional stimuli (for example, [30,74]) and the integration of affective arousal responses with the perception of current physiological states [75]. Although several studies found a differential activation between SAD patients and controls in the insula (for example, [5,8,10,63,66]) others did not (for example, [3,6,13,60,61]). The delayed emergence of insula hyperactivation in SAD patients in the present study might indicate an increased monitoring of bodily states that follows after an initial phase of arousal and hypervigilance during the confrontation with disorder-related video clips. Bodily responses might in turn be monitored in more detail and assessed as well as integrated into cached models of physiological response patterns and stimulus related autobiographic and declarative information about the particular threat. These processes were proposed to contribute to the maintenance of social anxiety [76].

The response pattern of anterior dorsal ACC supports previous findings of increased activation in medial prefrontal cortex areas in response to threatening stimuli or situations in patients with anxiety disorders [32], including SAD patients (for example, [6,8,66], but see [19,37,62]). Our results suggest a time-independent, constant affective-cognitive processing of threat in SAD due to the assumed role of midline regions of prefrontal cortex. This may reflect the special characteristics of the video stimuli used in the present study, but it might in part also indicate greater self-referential and self-regulative processes [23-25] in SAD patients. Generally, individuals suffering from SAD are excessively self-focused [76], which may strongly rely on prefrontal functions [21,77-79]. Heightened self-focused attention seems to cause exaggerated negative self-evaluation, anxiety and arousal, and even social withdrawal [80] and is therefore a potentially relevant mediator for the development and maintenance of SAD.

We would like to note several limitations of our study. We decided to analyze the video-related time courses based on a split-half method and refrained from using finer-grained time scale resolutions for the sake of parsimony. Further studies should investigate the time course of different brain areas with higher temporal resolutions. Furthermore, additional analyses did not reveal significant correlations between enhanced brain activation in the ROIs and symptom severity in SAD patients, suggesting limited clinical relevance of the present findings. The lack of significant correlations might be due to BOLD ceiling effects in SAD during processing of social video clips or varying effectiveness of different categories of video clips for different patients. These points should be investigated with increased sample sizes. Finally, we investigated only one method of symptom provocation. Our findings might be restricted to the stimuli used here. Future studies should compare different methods of symptom provocation in order to investigate whether similar effects are also present with other designs. Nevertheless, our results suggest that responses in the amygdala, the insula, and other areas might be associated with a specific time course during symptom provocation.

## Conclusions

In summary, using a newly developed symptom provocation design, we found different phases of brain activation in SAD patients as compared to controls when exposed to disorder-related and anxiety-provoking *versus* neutral video clips. We found increased amygdala activation during the first half of the video clips and increased insula activation during the second half in SAD patients compared to controls. Activation in medial prefrontal areas was significantly enhanced during the whole exposure period. Our findings support the prominent role of the amygdala in a transient threat detection system and the importance of the insula for prolonged and sustained processing of threat, while the time invariant hyperactivation pattern of anterior dorsal ACC is in accordance with current cognitive models of SAD.

## Abbreviations

ANOVA: Analysis of variance; BDI: Beck depression inventory; BOLD: Blood oxygen-level-dependent; ACC: Anterior cingulate cortex; dmPFC: Dorsomedial prefrontal cortex; DSM-IV-TR: Diagnostic and statistical manual of mental disorders, 4. Ed., text revision; fMRI: Functional magnetic resonance imaging; GLM: General linear model; HC: Healthy control; LSAS: Liebowitz social anxiety scale; ROI: Region of interest; SAD: Social anxiety disorder; SCID: Structured clinical interview for DSM-IV; TE: Echo time; TR: Repetition time.

## Competing interests

The authors declare that they have no competing interests.

## Authors’ contributions

SB participated in data collection, data preprocessing, performed the statistical analysis, and drafted the manuscript. AM participated in the design of the study, conducted the study, and was involved in data preprocessing and analysis. MPIB helped in the analysis of the data and to draft the manuscript. WHRM participated in the design of the study and helped to draft the manuscript. TS participated in the design and analysis of the study and drafted the manuscript. All authors read and approved the final manuscript.

## Supplementary Material

Additional file 1: Table S1 Description of the used video clips. Click here for file

## References

[B1] American Psychiatric Association Diagnostic and statistical manual of mental disorders (DSM-IV-TR) 2000 Washington, DC: APA

[B2] Gentili C Ricciardi E Gobbini MI Santarelli MF Haxby JV Pietrini P Guazzelli M Beyond amygdala: default mode network activity differs between patients with social phobia and healthy controls Brain Res Bull 2009 4 409 413 10.1016/j.brainresbull.2009.02.002 19559343

[B3] Yoon KL Fitzgerald DA Angstadt M McCarron RA Phan KL Amygdala reactivity to emotional faces at high and low intensity in generalized social phobia: a 4-Tesla functional MRI study Psychiatry Res 2007 4 93 98 10.1016/j.pscychresns.2006.05.004 17097275

[B4] Blair K Geraci M Devido J McCaffrey D Chen G Vythilingam M Ng P Hollon N Jones M Blair RJ Pine DS Neural response to self- and other referential praise and criticism in generalized social phobia Arch Gen Psychiatry 2008 4 1176 1184 10.1001/archpsyc.65.10.1176 18838634 PMC2785901

[B5] Straube T Mentzel H Miltner W Common and distinct brain activation to threat and safety signals in social phobia Neuropsychobiology 2005 4 163 168 10.1159/000087987 16137995

[B6] Phan KL Fitzgerald DA Nathan PJ Tancer ME Association between amygdala hyperactivity to harsh faces and severity of social anxiety in generalized social phobia Biol Psychiatry 2006 4 424 429 10.1016/j.biopsych.2005.08.012 16256956

[B7] Schmidt S Mohr A Miltner WHR Straube T Task-dependent neural correlates of the processing of verbal threat-related stimuli in social phobia Biol Psychol 2010 4 304 312 10.1016/j.biopsycho.2010.03.005 20227458

[B8] Straube T Kolassa IT Glauer M Mentzel H Miltner W Effect of task conditions on brain responses to threatening faces in social phobics: an event-related functional magnetic resonance imaging study Biol Psychiatry 2004 4 921 930 10.1016/j.biopsych.2004.09.024 15601601

[B9] Schulz C Mothes-Lasch M Straube T Automatic neural processing of disorder-related stimuli in social anxiety disorder (SAD): faces and more Front Psychol 2013 4 282 23745116 10.3389/fpsyg.2013.00282 PMC3662886

[B10] Boehme S Ritter V Tefikow S Stangier U Strauss B Miltner WHR Straube T Brain activation during anticipatory anxiety in social anxiety disorder Soc Cogn Affect Neurosci 2013 [Epub ahead of print] 10.1093/scan/nst129 PMC4158379 23938870

[B11] Furmark T Appel L Henningsson S Åhs F Faria V Linnman C Pissiota A Frans Ö Bani M Bettica P Pich EM Jacobbson E Wahlstedt K Oreland L Langstrom B Eriksson E Fredrikson M A link between serotonin-related gene polymorphisms, amygdala activity, and placebo-induced relief from social anxiety J Neurosci 2008 4 13066 13074 10.1523/JNEUROSCI.2534-08.2008 19052197 PMC6671592

[B12] Furmark T Appel L Michelgard A Wahlstedt K Ahs F Zancan S Jacobsson E Flyckt K Grohp M Bergstrom M Pich EM Nilsson LG Bani M Langstrom B Fredrikson M Cerebral blood flow changes after treatment of social phobia with the neurokinin-1 antagonist GR205171, citalopram, or placebo Biol Psychiatry 2005 4 132 142 10.1016/j.biopsych.2005.03.029 16038684

[B13] Tillfors M Furmark T Marteinsdottir I Fischer H Pissiota A Langstrom B Fredrikson M Cerebral blood flow in subjects with social phobia during stressful speaking tasks: a PET study Am J Psychiatry 2001 4 1220 1226 10.1176/appi.ajp.158.8.1220 11481154

[B14] Furmark T Tillfors M Marteinsdottir I Fischer H Pissiota A Langstrom B Fredrikson M Common changes in cerebral blood flow in patients with social phobia treated with citalopram or cognitive-behavioral therapy Arch Gen Psychiatry 2002 4 425 433 10.1001/archpsyc.59.5.425 11982446

[B15] Öhman A The role of the amygdala in human fear: automatic detection of threat Psychoneuroendocrinology 2005 4 953 958 10.1016/j.psyneuen.2005.03.019 15963650

[B16] LeDoux J The emotional brain, fear, and the amygdala Cell Mol Neurobiol 2003 4 727 738 10.1023/A:1025048802629 14514027 PMC11530156

[B17] Phan KL Fitzgerald DA Cortese BM Seraji-Bozorgzad N Tancer ME Moore GJ Anterior cingulate neurochemistry in social anxiety disorder: 1H-MRS at 4 Tesla Neuroreport 2005 4 183 186 10.1097/00001756-200502080-00024 15671874

[B18] Campbell DW Sareen J Paulus MP Goldin PR Stein MB Reiss JP Time-varying amygdala response to emotional faces in generalized social phobia Biol Psychiatry 2007 4 455 463 10.1016/j.biopsych.2006.09.017 17188251

[B19] Blair KS Geraci M Smith BW Hollon N DeVido J Otero M Blair JR Pine DS Reduced dorsal anterior cingulate cortical activity during emotional regulation and top-down attentional control in generalized social phobia, generalized anxiety disorder, and comorbid generalized social phobia/generalized anxiety disorder Biol Psychiatry 2012 4 476 482 10.1016/j.biopsych.2012.04.013 22592057 PMC3424322

[B20] Goldin PR Manber T Hakimi S Canli T Gross JJ Neural bases of social anxiety disorder: emotional reactivity and cognitive regulation during social and physical threat Arch Gen Psychiatry 2009 4 170 180 10.1001/archgenpsychiatry.2008.525 19188539 PMC4142809

[B21] Pessoa L On the relationship between emotion and cognition Nat Rev Neurosci 2008 4 148 158 10.1038/nrn2317 18209732

[B22] Etkin A Egner T Peraza DM Kandel ER Hirsch J Resolving emotional conflict: a role for the rostral anterior cingulate cortex in modulating activity in the amygdala Neuron 2006 4 871 882 10.1016/j.neuron.2006.07.029 16982430

[B23] Kalisch R Wiech K Critchley HD Dolan RJ Levels of appraisal: a medial prefrontal role in high-level appraisal of emotional material Neuroimage 2006 4 1458 1466 10.1016/j.neuroimage.2005.11.011 16388969

[B24] Phan KL Wager T Taylor SF Liberzon I Functional neuroanatomy of emotion: a meta-analysis of emotion activation studies in PET and fMRI Neuroimage 2002 4 331 348 10.1006/nimg.2002.1087 12030820

[B25] Ochsner KN Beer JS Robertson ER Cooper JC Gabrieli JDE Kihsltrom JF D’Esposito M The neural correlates of direct and reflected self-knowledge NeuroImage 2005 4 797 814 10.1016/j.neuroimage.2005.06.069 16290016

[B26] Northoff G Heinzel A de Greck M Bermpohl F Dobrowolny H Panksepp J Self-referential processing in our brain–a meta-analysis of imaging studies on the self NeuroImage 2006 4 440 457 10.1016/j.neuroimage.2005.12.002 16466680

[B27] Craig AD How do you feel? Interoception: the sense of the physiological condition of the body Nat Rev Neurosci 2002 4 655 666 12154366 10.1038/nrn894

[B28] Critchley HD Mathias CJ Josephs O O’Doherty J Zanini S Dewar BK Cipolotti L Shallice T Dolan RJ Human cingulate cortex and autonomic control: converging neuroimaging and clinical evidence Brain 2003 4 2139 2152 10.1093/brain/awg216 12821513

[B29] Critchley HD Wiens S Rotshtein P Ohman A Dolan RJ Neural systems supporting interoceptive awareness Nat Neurosci 2004 4 189 195 10.1038/nn1176 14730305

[B30] Critchley HD The human cortex responds to an interoceptive challenge Proc Natl Acad Sci U S A 2004 4 6333 6334 10.1073/pnas.0401510101 15096592 PMC404044

[B31] Straube T Miltner WHR Attention to aversive emotion and specific activation of the right insula and right somatosensory cortex NeuroImage 2011 4 2534 2538 10.1016/j.neuroimage.2010.10.010 20946962

[B32] Etkin A Wager TD Functional neuroimaging of anxiety: a meta-analysis of emotional processing in PTSD, social anxiety disorder, and specific phobia Am J Psychiatry 2007 4 1476 1488 10.1176/appi.ajp.2007.07030504 17898336 PMC3318959

[B33] Sladky R Höflich A Atanelov J Kraus C Baldinger P Moser E Lanzenberger R Windischberger C Increased neural habituation in the amygdala and orbitofrontal cortex in social anxiety disorder revealed by fMRI PLoS One 2012 4 e50050 10.1371/journal.pone.0050050 23209643 PMC3510234

[B34] Åhs F Furmark T Michelgård Å Långström B Appel L Wolf OT Kirschbaum C Fredrikson M Hypothalamic blood flow correlates positively with stress-induced cortisol levels in subjects with social anxiety disorder Psychosom Med 2006 4 859 862 10.1097/01.psy.0000242120.91030.d8 17079706

[B35] Ziv M Goldin P Jazaieri H Hahn K Gross J Is there less to social anxiety than meets the eye? behavioral and neural responses to three socio-emotional tasks Biol Mood Anxiety Disord 2013 4 5 10.1186/2045-5380-3-5 23448192 PMC3608942

[B36] Kilts CD Kelsey JE Knight B Ely TD Bowman FD Gross RE Selvig A Gordon A Newport DJ Nemeroff CB The neural correlates of social anxiety disorder and response to pharmacotherapy Neuropsychopharmacology 2006 4 2243 2253 16525417 10.1038/sj.npp.1301053

[B37] Van Ameringen M Mancini C Szechtman H Nahmias C Oakman JM Hall GBC Pipe B Farvolden P A PET provocation study of generalized social phobia Psychiatry Res Neuroimaging 2004 4 13 18 10.1016/j.pscychresns.2004.07.005 15546699

[B38] Furmark T Social phobia: overview of community surveys Acta Psychiatr Scand 2002 4 84 93 10.1034/j.1600-0447.2002.1r103.x 11939957

[B39] Wendt J Lotze M Weike A Hosten N Hamm A Brain activation and defensive response mobilization during sustained exposure to phobia-related and other affective pictures in spider phobia Psychophysiology 2008 4 205 215 10.1111/j.1469-8986.2007.00620.x 17995911

[B40] Wendt J Schmidt LE Lotze M Hamm AO Mechanisms of change: effects of repetitive exposure to feared stimuli on the brain’s fear network Psychophysiology 2012 4 1319 1329 10.1111/j.1469-8986.2012.01451.x 22913381

[B41] Wright CI Fischer H Whalen PJ McInerney SC Shin LM Rauch SL Differential prefrontal cortex and amygdala habituation to repeatedly presented emotional stimuli NeuroReport 2001 4 379 383 10.1097/00001756-200102120-00039 11209954

[B42] Straube T Mentzel H Miltner W Neural mechanisms of automatic and direct processing of phobogenic stimuli in specific phobia Biol Psychiatry 2006 4 162 170 10.1016/j.biopsych.2005.06.013 16139812

[B43] Stein MB Simmons AN Feinstein JS Paulus MP Increased amygdala and insula activation during emotion processing in anxiety-prone subjects Am J Psychiatry 2007 4 318 327 10.1176/appi.ajp.164.2.318 17267796

[B44] Rauch SL Savage CR Alpert NM Fischman AJ Jenike MA The functional neuroanatomy of anxiety: a study of three disorders using positron emission tomography and symptom provocation Biol Psychiatry 1997 4 446 452 10.1016/S0006-3223(97)00145-5 9285080

[B45] Feinstein JS Adolphs R Damasio A Tranel D The human amygdala and the induction and experience of fear Curr Biol 2011 4 34 38 10.1016/j.cub.2010.11.042 21167712 PMC3030206

[B46] Rottenberg J Ray RD Gross JJ Coan JA, Allen JJB Emotion elicitation using films Handbook of emotion elicitation and assessment 2007 New York: Oxford University Press 9 28

[B47] Gerrards-Hesse A Spies K Hesse FW Experimental inductions of emotional states and their effectiveness: a review Br J Psychol 1994 4 55 78 10.1111/j.2044-8295.1994.tb02508.x

[B48] Fydrich T Renneberg B Schmitz B Wittchen HU Strukturiertes klinisches interview für DSM-IV, Achse II (Persönlichkeitsstörungen) 1997 Göttingen: Hogrefe

[B49] Wittchen H-U Wunderlich U Gruschwitz S Zaudig M Strukturiertes klinisches interview für DSM-IV, Achse-I (SKID-I) 1997 Göttingen: Hogrefe

[B50] Stangier U Heidenreich T Scalarum CIP Liebowitz social anxiety scale Internationale Skalen für psychiatrie (Internatioal scales for psychiatry) 2005 Weinheim: Beltz 299 306

[B51] Hautzinger M Bailer M Worall H Keller F Beck-depressions-inventar (BDI). Testhandbuch der deutschen Ausgabe 1995 Bern: Huber

[B52] Talairach J Tournoux P Co-planar stereotaxic atlas of the human brain: 3-dimensional proportional system: an approach to cerebral imaging 1988 Stutgart: Thieme

[B53] Eickhoff SB Heim S Zilles K Amunts K Testing anatomically specified hypotheses in functional imaging using cytoarchitectonic maps NeuroImage 2006 4 570 582 10.1016/j.neuroimage.2006.04.204 16781166

[B54] Eickhoff SB Stephan KE Mohlberg H Grefkes C Fink GR Amunts K Zilles K A new SPM toolbox for combining probabilistic cytoarchitectonic maps and functional imaging data NeuroImage 2005 4 1325 1335 10.1016/j.neuroimage.2004.12.034 15850749

[B55] Maldjian JA Laurienti PJ Burdette JH Precentral gyrus discrepancy in electronic versions of the Talairach atlas NeuroImage 2004 4 450 455 10.1016/j.neuroimage.2003.09.032 14741682

[B56] Maldjian JA Laurienti PJ Kraft RA Burdette JH An automated method for neuroanatomic and cytoarchitectonic atlas-based interrogation of fMRI data sets NeuroImage 2003 4 1233 1239 10.1016/S1053-8119(03)00169-1 12880848

[B57] Tzourio-Mazoyer N Landeau B Papathanassiou D Crivello F Etard O Delcroix N Mazoyer B Joliot M Automated anatomical labeling of activations in SPM using a macroscopic anatomical parcellation of the MNI MRI single-subject brain NeuroImage 2002 4 273 289 10.1006/nimg.2001.0978 11771995

[B58] Lancaster JL Tordesillas-Gutiérrez D Martinez M Salinas F Evans A Zilles K Mazziotta JC Fox PT Bias between MNI and Talairach coordinates analyzed using the ICBM-152 brain template Hum Brain Mapp 2007 4 1194 1205 10.1002/hbm.20345 17266101 PMC6871323

[B59] Forman SD Cohen JD Fitzgerald M Eddy WF Mintun MA Noll DC Improved assessment of significant activation in functional magnetic resonance imaging (fMRI): use of a cluster-size threshold Magn Reson Med 1995 4 636 647 10.1002/mrm.1910330508 7596267

[B60] Cooney RE Atlas LY Joormann J Eugene F Gotlib IH Amygdala activation in the processing of neutral faces in social anxiety disorder: is neutral really neutral? Psychiatry Res 2006 4 55 59 10.1016/j.pscychresns.2006.05.003 17030117

[B61] Stein M Goldin P Sareen J Zorrilla L Brown G Increased amygdala activation to angry and contemptuous faces in generalized social phobia Arch Gen Psychiatry 2002 4 1027 1034 10.1001/archpsyc.59.11.1027 12418936

[B62] Gentili C Gobbini MI Ricciardi E Vanello N Pietrini P Haxby JV Guazzelli M Differential modulation of neural activity throughout the distributed neural system for face perception in patients with social phobia and healthy subjects Brain Res Bull 2008 4 286 292 10.1016/j.brainresbull.2008.08.003 18771714

[B63] Shah SG Klumpp H Angstadt M Nathan PJ Luan Phan K Amygdala and insula response to emotional images in patients with generalized social anxiety disorder 2009 Ottawa, ON: Canadian Medical Association PMC2702447 19568481

[B64] Blair KS Geraci M Otero M Majestic C Odenheimer S Jacobs M Blair RJR Pine DS Atypical modulation of medial prefrontal cortex to self-referential comments in generalized social phobia Psychiatry Res Neuroimaging 2011 4 38 45 10.1016/j.pscychresns.2010.12.016 PMC3105197 21601433

[B65] Klumpp H Angstadt M Phan KL Insula reactivity and connectivity to anterior cingulate cortex when processing threat in generalized social anxiety disorder Biol Psychol 2012 4 273 276 10.1016/j.biopsycho.2011.10.010 22027088 PMC3260042

[B66] Amir N Klumpp H Elias J Bedwell JS Yanasak N Miller LS Increased activation of the anterior cingulate cortex during processing of disgust faces in individuals with social phobia Biol Psychiatry 2005 4 975 981 10.1016/j.biopsych.2005.01.044 15860337

[B67] Quadflieg S Mohr A Mentzel H Miltner W Straube T Modulation of the neural network involved in the processing of anger prosody: the role of task-relevance and social phobia Biol Psychol 2008 4 129 137 10.1016/j.biopsycho.2008.01.014 18353521

[B68] Tamietto M de Gelder B Neural bases of the non-conscious perception of emotional signals Nat Rev Neurosci 2010 4 697 709 20811475 10.1038/nrn2889

[B69] LeDoux J Emotion circuits in the brain Annu Rev Neurosci 2000 4 155 184 10.1146/annurev.neuro.23.1.155 10845062

[B70] Pessoa L Adolphs R Emotion processing and the amygdala: from a ‘low road’ to ‘many roads’ of evaluating biological significance Nat Rev Neurosci 2010 4 773 783 20959860 10.1038/nrn2920 PMC3025529

[B71] LeDoux J Fear and the brain: where have we been, and where are we going? Biol Psychiatry 1998 4 1229 1238 10.1016/S0006-3223(98)00282-0 9861466

[B72] Öhman A Mineka S Fears, phobias, and preparedness: toward an evolved module of fear and fear learning Psychol Rev 2001 4 483 522 11488376 10.1037/0033-295x.108.3.483

[B73] Lipka J Miltner W Straube T Vigilance for threat interacts with amygdala responses to subliminal threat cues in specific phobia Biological Psychiatry 2011 2011 4 472 478 10.1016/j.biopsych.2011.04.005 21601831

[B74] Damasio AR Grabowski TJ Bechara A Damasio H Ponto LL Parvizi J Hichwa RD Subcortical and cortical brain activity during the feeling of self-generated emotions Nat Neurosci 2000 4 1049 1056 10.1038/79871 11017179

[B75] Craig AD How do you feel – now? The anterior insula and human awareness Nat Rev Neurosci 2009 4 59 70 10.1038/nrn2555 19096369

[B76] Clark DM Wells A Heimberg RG, Liebowitz MR, Hope DA, Schneier FR A cognitive model of social phobia Social phobia: diagnosis, assessment, and treatment 1995 New York: Guilford Press 69 93

[B77] Northoff G Bermpohl F Cortical midline structures and the self Trends Cogn Sci 2004 4 102 107 10.1016/j.tics.2004.01.004 15301749

[B78] van der Meer L Costafreda S Aleman A David AS Self-reflection and the brain: a theoretical review and meta-analysis of neuroimaging studies with implications for schizophrenia Neurosci Biobehav Rev 2010 4 935 946 10.1016/j.neubiorev.2009.12.004 20015455

[B79] Mitchell JP Banaji MR Macrae CN The link between social cognition and self-referential thought in the medial prefrontal cortex J Cogn Neurosci 2005 4 1306 1315 10.1162/0898929055002418 16197685

[B80] Clark DM McManus F Information processing in social phobia Biol Psychiatry 2002 4 92 100 10.1016/S0006-3223(01)01296-3 11801234

