# Supplementary material for: Area-dependent time courses of brain activation during video-induced symptom provocation in social anxiety disorder
Source: Biol Mood Anxiety Disord. 2014 Apr 28;4:6. doi: 10.1186/2045-5380-4-6 (PMC4052290; doi:10.1186/2045-5380-4-6)
Supplement: Additional file 1: Table S1 — Description of the used video clips. [file 2045-5380-4-6-S1.docx]

# Supplementary Material

Tab. S1: Description of the used video clips.

|  |  | disorder relevant | neutral |
| --- | --- | --- | --- |
| *formal interaction situations* | | |  |
|  | 1 | A woman speaks in front of an audience with a microphone. | The woman leaves the empty lecture room. |
|  | 2 | A man knocks on the Professor's door and enters the examination room. | The man walks along the floor and randomly reads the postings. |
|  | 3 | A man speaks into a microphone to an audience (from speaker’s perspective). | The man walks through a public building, looks at a floor plan and continues to walk. |
|  | 4 | A woman gives a talk in a crowded seminar room. | The woman cleans the table in the empty seminar room. |
|  | 5 | A woman is presenting and discussing something at a business meeting. | The woman sits alone in her office and works. |
| *informal interaction situations* | | |  |
|  | 1 | A woman asks a stranger for directions. | The woman walks along an empty street, looks in a map and continues to walk. |
|  | 2 | A woman and a man sit closely at a table with two glasses of wine, smile, and talk to each other. | The woman sits at the table alone with a glass of wine and reads a book. |
|  | 3 | A man stands among other persons in a crowded elevator. | The man stands alone in the elevator. |
|  | 4 | A man carrying a tray walks through a crowded canteen and looks for a free seat. | The man clears away his tray in the empty canteen. |
| *situations that require self-assurance* | | |  |
|  | 1 | A man arrives at his car and finds two traffic wardens writing a parking ticket. The man addresses the wardens and argues with them. | The man approaches his car and gets in. |
|  | 2 | A woman knocks on her professor’s door and enters the room where a meeting is about to take place. | The woman walks through the building and takes a look at the postings. |
|  | 3 | A man sits in front of a door and waits. A man steps out, shows a piece of paper to the waiting person and shakes his head in dissatisfaction. | The man randomly takes a seat in the hallway and starts to read exhibited information material. |
|  | 4 | At a store, a woman complains about a pair of shoes she has bought. | The woman looks at shoes alone in a corner of the store. |
| *situations where the actor’s behavior is observed by others* | | |  |
|  | 1 | A woman is sitting in front of her computer. A man standing next to her is supervising her typing and urges her to hurry up, pointing at his watch. | The woman sits alone in the office in front of her computer. |
|  | 2 | A man arrives late for a seminar and enters the room after the talk has begun. | The man leaves the empty seminar room. |
|  | 3 | A man makes a telephone call in a crowded open-plan office. | The man sits in the empty open-plan office and works. |
|  | 4 | A man sits at a lunch table in a crowded canteen, eats and talks to peers. | The man makes coffee alone in the kitchen. |
|  | 5 | A woman sits in a restaurant with her peers when her mobile phone starts to ring. Keeping her seat, she answers the call. | The woman sits alone in the restaurant drinking tea and reading a magazine. |
|  |  |  |  |
